# The effect of vitamin K2 supplementation on bone turnover biochemical markers in postmenopausal osteoporosis patients: a systematic review and meta-analysis

**DOI:** 10.3389/fendo.2025.1703116

**Published:** 2025-11-05

**Authors:** Zechen Zhang, Yuyi Li, Jinkun Li, Yifeng Yuan, Kang Liu, Xiaolin Shi

**Affiliations:** ^1^ The Second School of Clinical Medicine, Zhejiang Chinese Medical University, Hangzhou, China; ^2^ The Second Affiliated Hospital of Zhejiang Chinese Medical University (Xinhua Hospital of Zhejiang Province), Hangzhou, China

**Keywords:** vitamin K2, osteoporosis, bone turnover markers, meta-analysis, osteocalcin, ucOC, bone metabolism

## Abstract

**Background:**

Osteoporosis is a metabolic bone disease characterized by decreased bone mass and increased fracture risk. Bone turnover markers, such as osteocalcin (OC), undercarboxylated osteocalcin (ucOC), and other biochemical indicators, are important for assessing bone metabolism. Vitamin K2 influences bone metabolism by enhancing osteocalcin γ-carboxylation.

**Methods:**

This study followed PRISMA guidelines and included randomized controlled trials on the effects of vitamin K2 supplementation on bone turnover biomarkers in postmenopausal osteoporosis patients. Key outcomes included changes in OC, ucOC, and other bone metabolism markers.

**Results:**

Nine studies with 2,570 participants were included. Vitamin K2 (VK2) increased osteocalcin (OC; MD 1.86, 95% CI 1.17–2.56) and bone-specific alkaline phosphatase (BAP; MD 1.49, 95% CI 0.98–2.00). It reduced undercarboxylated OC (ucOC; WMD −1.54, 95% CI −2.44 to −0.64) and tartrate-resistant acid phosphatase (TRAP; MD −0.83, 95% CI −1.21 to −0.46). C-terminal telopeptide (CTX) showed a small, statistically significant reduction (MD −0.09, 95% CI −0.14 to −0.05) with uncertain clinical relevance. N-telopeptide (NTX) showed no significant change.

**Conclusions:**

Vitamin K2 supplementation improves key bone turnover biomarkers, particularly OC and ucOC. These findings support its role in bone metabolism, though further long-term studies are needed to confirm clinical benefits, such as increased bone mineral density.

**Systematic review registration:**

https://www.crd.york.ac.uk/PROSPERO/view/CRD420251087067, identifier CRD420251087067.

## 1 Introduction

Osteoporosis is a common metabolic bone disease. It is characterized by low bone mass and deterioration of bone microarchitecture. These changes increase fracture risk. A meta-analysis of 86 studies with 103 million participants estimated a global prevalence of 18.3% (women 23.1%, men 11.7%) (1). Fractures cause disability, hospitalizations, and functional decline. They also increase premature mortality and create a major socioeconomic burden. In the Global Burden of Disease 2019 analysis, low bone mineral density was associated with about 438,000 deaths and 166 million disability-adjusted life years, with large increases since 1990 (2).

Bone remodeling depends on the balance between formation and resorption. Core formation markers include bone-specific alkaline phosphatase (BAP; also termed BSALP), procollagen type I N-terminal propeptide (P1NP), and total osteocalcin (OC). Resorption markers include type I collagen C-telopeptide (CTX) and N-telopeptide (NTX) (3). These dynamic indicators help assess turnover rates and monitor treatment efficacy (4). Vitamin K–related osteocalcin indices—including undercarboxylated osteocalcin (ucOC), carboxylated osteocalcin (cOC), and the ucOC/OC ratio—are useful indicators of both vitamin K status and bone quality. In a cross-sectional study of 900 Chinese adults, higher ucOC was associated with lower BMD at the spine, femoral neck, and hip, and with higher P1NP and β-CTX, indicating increased turnover (5).

Vitamin K exists as phylloquinone (K1) and menaquinones (K2). K1 is abundant in green leafy vegetables but has low intestinal absorption (≈5–10%). Long-chain K2 forms, especially MK-7, show higher bioavailability and a longer half-life, supporting extrahepatic actions such as bone metabolism (6). Beyond coagulation, K2 contributes to calcium homeostasis. K2 deficiency has been linked to the “calcium paradox,” with insufficient skeletal deposition and increased vascular calcification (7). Some studies suggest that K2, alone or combined with vitamin D, may support bone health in postmenopausal women by reducing bone loss and improving strength (8). Mechanistically, K2 acts as a cofactor for γ-glutamyl carboxylase and supports the vitamin K cycle, enabling γ-carboxylation of osteocalcin and other vitamin K–dependent proteins (9–11). Menaquinones may also activate SXR/PXR signaling, enhance osteoblast differentiation and matrix synthesis, and up-regulate OPG while dampening RANKL/NF-κB pathways, thereby reducing osteoclast activity (12–14).

Clinical findings on K2 (particularly MK-4 and MK-7) for BMD and fractures are inconsistent. Pharmacologic-dose MK-4 reduced fractures in some Japanese cohorts, whereas low-dose MK-7 yielded site-specific effects in some trials and null results in others; studies outside Japan often showed no significant benefits for BMD or fractures (13, 15–19). These discrepancies likely reflect differences in dose, co-supplementation, baseline vitamin K status, and study populations (13, 18, 19). Compared with hard endpoints, biochemical markers respond earlier. K2 consistently lowers ucOC and the ucOC/OC ratio within months (13, 15, 20, 21). International consensus supports serum P1NP and β-CTX as reference standards for monitoring osteoporosis treatment, underscoring the value of bone turnover markers as dynamic outcomes (22, 23). However, focused evidence syntheses on BTMs remain limited; many reviews emphasize BMD and fractures and treat biochemical outcomes as secondary. This gap motivates a targeted evaluation.

We therefore conducted a systematic review and meta-analysis of randomized controlled trials to evaluate the effects of vitamin K2 on bone turnover biomarkers in postmenopausal osteoporosis.

## 2 Methods

This study has been registered with the International Prospective Register of Systematic Reviews (PROSPERO registration number: CRD420251087067). The reporting of this study follows the PRISMA (Preferred Reporting Items for Systematic Reviews and Meta-Analyses) guidelines.

### 2.1 Inclusion and exclusion criteria

Eligibility criteria were defined using the PICO-S framework: Participants (P) were postmenopausal women with a definitive diagnosis of osteoporosis, defined by a bone mineral density (BMD) T-score ≤ −2.5 SD at the lumbar spine, total hip, or femoral neck and/or a history of fragility fracture. Intervention (I) consisted of vitamin K2 supplementation in any form (e.g., MK-4, MK-7) or dosage. Co-interventions such as calcium, vitamin D, or other medications were permitted provided the control group received identical concomitant treatments excluding vitamin K2. The Comparator (C) comprised other active medications, placebo, or calcium/vitamin D supplementation without vitamin K2, ensuring the control group received the same baseline treatment as the intervention group minus the vitamin K2 component. Primary Outcomes (O) focused on changes in serum/plasma bone turnover biomarkers (post-intervention vs. baseline or intervention vs. control group end values/change scores), specifically: Vitamin K status-related markers: undercarboxylated osteocalcin (ucOC), carboxylated osteocalcin (cOC), ucOC/Total OC ratio, ucOC/cOC ratio; Bone formation markers: Total osteocalcin (Total OC), bone-specific alkaline phosphatase (BAP), procollagen type I N-terminal propeptide (P1NP); and Bone resorption markers: C-terminal telopeptide of type I collagen (CTX), N-terminal telopeptide of type I collagen (NTX), etc. Study design (S) was restricted to randomized controlled trials (RCTs).

Studies were excluded if they: (1) had insufficient published data and the corresponding author did not respond to requests for further information; (2) were single-arm studies including only one group; (3) were conference abstracts, experimental studies, or basic research.

### 2.2 Literature search strategy

A comprehensive literature search was conducted across PubMed, Cochrane Library, EMBASE, Web of Science, CNKI, Wanfang, and VIP databases from the inception of the databases to July 6, 2025. A combination of MeSH terms and free text words was used for the search. The keywords included “ Vitamin K2”, “ Menaquinone “, “ menatetrenone “, “ Bone Density “, “osteoporosis”, “Osteoporotic Fractures”, “ Fractures, Bone “. The detailed search process is provided in the **Supplementary Materials**. No restrictions on study type were applied during the literature search.

No language restrictions were applied to the search. Outcome terms for bone turnover markers were not used at the search stage to avoid missing trials that reported BTMs only in the full text or as secondary outcomes; BTM eligibility was applied during screening and data extraction (OC, cOC/ucOC, BAP/BSALP, P1NP, CTX/β-CTX, NTX, TRAP).

### 2.3 Research screening and selection

Records were managed and deduplicated using the EndNote 20 reference management software. The screening was conducted independently by two researchers in two stages. First, an initial screening was performed based on titles and abstracts. Subsequently, the full texts of potentially eligible records were retrieved and independently assessed against the predefined inclusion and exclusion criteria by both researchers. Any disagreements arising during screening or eligibility assessment were resolved through discussion between the two reviewers or, if necessary, by consultation with a third researcher. The entire screening process, including the number of records at each stage and the specific reasons for exclusion, was documented using a PRISMA flow diagram.

### 2.4 Data extraction

Two researchers independently extracted the following data using a standardized form, with cross-verification and resolution of discrepancies through discussion or third-party adjudication: First author, publication year, country, study duration, conflict of interest statements, Sample size (intervention/control groups), age, sex. Interventions: Vitamin K2 Group: Form, dosage, frequency of administration, treatment duration. Concomitant Treatments: Identical treatments received by both intervention and control groups. Control Group: Description of the comparator and any identical baseline treatment, outcome measures.

### 2.5 Risk of bias assessment

We assessed the risk of bias in randomized trials using the Cochrane RoB 2 tool across five domains (randomization, deviations from intended interventions, missing outcome data, outcome measurement, and selection of the reported result). Judgments were outcome-specific for the intention-to-treat (effect of assignment to intervention) target effect. Two reviewers assessed studies independently; disagreements were resolved by discussion with a third reviewer. We visualized the assessments with a RoB 2 traffic-light summary plot in the main text and a domain-level stacked percentage chart in the **Supplementary Material**. Overall judgments (low risk, some concerns, high risk) followed the RoB 2 decision algorithm.

### 2.6 Data synthesis and meta-analysis

For continuous outcomes (bone turnover biomarkers), the mean difference (MD) with 95% confidence interval (CI) will serve as the primary effect measure; however, the standardized mean difference (SMD) with 95% CI will be substituted when significant variations in measurement units or assay methods result in divergent outcome scales across studies. Model selection will follow quantitative heterogeneity thresholds: a random-effects model (DerSimonian and Laird method) will be applied where substantial heterogeneity is indicated by an I² statistic >50%, while a fixed-effect model will be employed for analyses demonstrating low-to-moderate heterogeneity (I² ≤50%). Publication bias assessment was performed only for the outcome that included the largest number of studies, using visual inspection of funnel plot asymmetry. Meta-analyses were conducted in Review Manager (RevMan) version 5.4, and figures (e.g., forest and funnel plots) were generated in RevMan.

## 3 Results

### 3.1 Literature search and screening results

**Figure 1:**
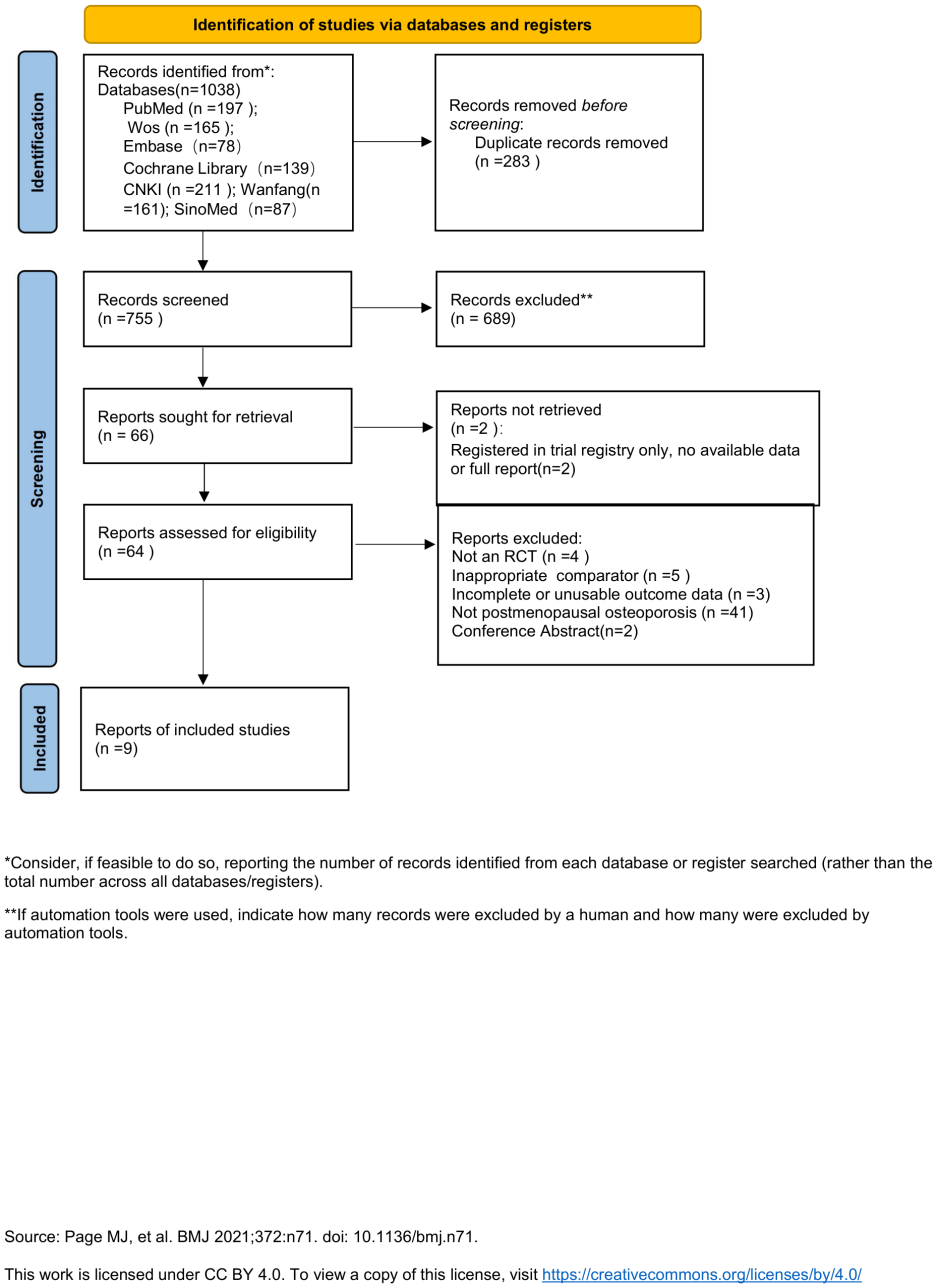
Flowchart of the selection process.

After comprehensive database searching, 1038 records related to vitamin K2 treatment for osteoporosis were retrieved. Following application of the eligibility criteria, 9 studies were ultimately included in this meta-analysis (24–32) (**Figure 1**).

### 3.2 Study characteristics

There were 9 studies with 2,570 participants, published between 2006 and 2022, predominantly from China and Japan. The mean participant age ranged from approximately 60 to 77 years. Treatment duration most commonly lasted 6 months.

Most studies employed combination therapy, where vitamin K2 was added to a base treatment of calcium and vitamin D, often alongside other bone-active drugs. Frequently reported outcome measures included osteocalcin (OC), bone alkaline phosphatase (BAP), and β-CTX. Funding sources were not reported, and the overall risk of bias was generally unclear (**Table 1**).

**Table 1:** The basic characteristics of included studies.

Reference	Sample size	T/C	Age(years)	Country	Intervention	Control	Treatment duration	Outcome measures
Gu et al (2019) (31)	210	70/70	64.07±9.63	China	Vitamin K_2_ + Control	Calcium + Vitamin D_3_ Chewable Tablets	6 months	BGP, β-Crosslaps, PINP, TRAP, Cathe K
Shiro et al (2016)	1874	931/943	T:75.3±5.8	Japan	Vitamin K_2_ + Control	Risedronate	2 years	uc-OC, 25(OH)-D
Yuji et al (2014)	101	51/50	T:75.4±5.7	Japan	Vitamin K_2_ + Control	Risedronate	2 years	NTX, BAP, Uc-OC, OC, Uc-OC/OC
Masataka et al (2009)	109	56/53	T:67.8±7.9	Japan	Vitamin K_2_	Calcium aspartate	6 months	OC, Gia-OC, UC-OC, BAP, U-NTX, U-DPD, S-25OHD, S-PTH
Yuditiya et al (2006)	63	33/30	T:60.9±4.9	Indonesia	Vitamin K_2_ + Control	Ca	48 weeks	UC-OC, OC
Zheng et al (2022) (24)	40	20/20	T:65.12±4.32	China	Vitamin K_2_ + Control	Calcium carbonate + Vitamin D_3_Tablets + Zoledronate	1 year	ALP, BGP
Wei et al (2022) (25)	51	26/25	T:76.42±6.33	China	Vitamin K_2_ + Control	Calcium carbonate + Vitamin D_3_ Granules + Alfacalcidol	6 months	OC
Xu et al (2021) (26)	40	20/20	T:73.7±4.3 C:73.2±4.2	China	Vitamin K_2_ + Control	Calcium carbonate + Vitamin D_3_ Tablets + Micapril	6 months	25(OH)D, OC, s-CTX, BAP, TRACP-5b
Fu et al (2020) (27)	82	41/41	T:71.5±15.6	China	Vitamin K_2_ + Control	Calcium carbonate + Vitamin D_3_ Tablets + Zoledronate	6 months	s-CTX, TRAP-5b, BAP, BGP

### 3.3 Risk of bias

**Figure 2:**
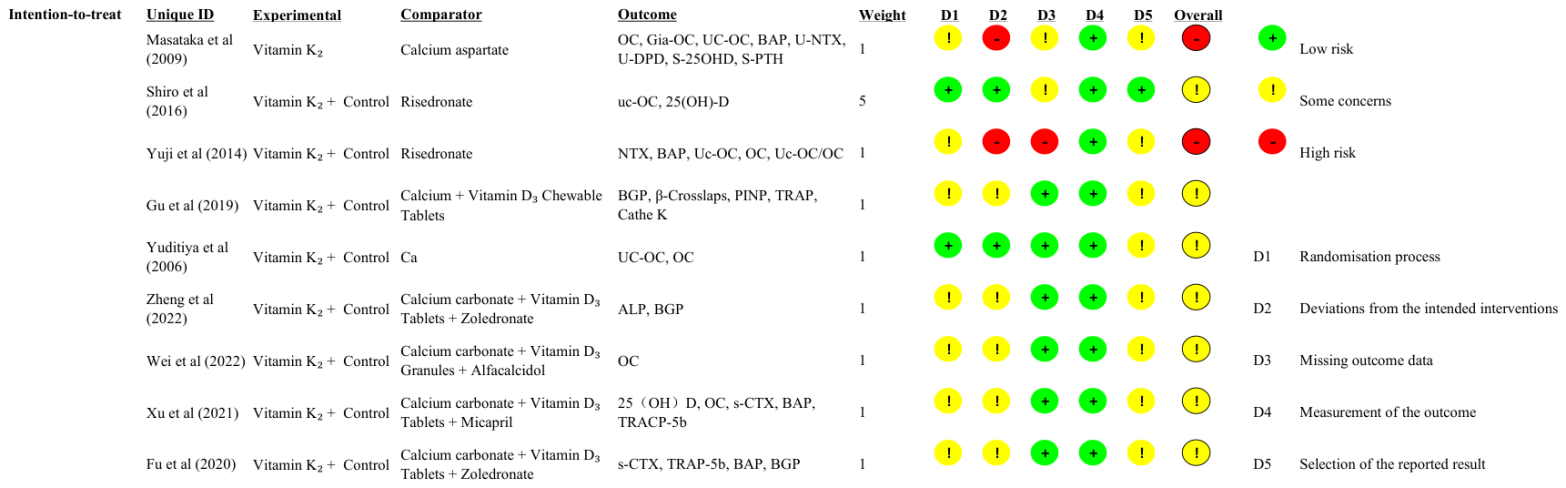
Risk-of-bias summary (RoB 2) for included randomized trials. Rows represent individual trials and columns correspond to the five RoB 2 domains: D1—bias arising from the randomization process; D2—bias due to deviations from intended interventions; D3—bias due to missing outcome data; D4—bias in measurement of the outcome; D5—bias in selection of the reported result. Colors denote judgment: green = low risk; yellow = some concerns; red = high risk; grey = no information. (An overall RoB 2 judgment is shown in the rightmost column.).

Domain-level judgments for all included trials are shown in **Figure 2** (green = low risk; yellow = some concerns; red = high risk; grey = no information). The distribution of these judgments across domains—summarized as percentages for the intention-to-treat target effect—is provided in **Supplementary Figure S1**, which also displays the spread of the overall RoB 2 judgments.

### 3.4 Meta-analysis results

#### 3.4.1 OC(ng/mL)

**Figure 3:**
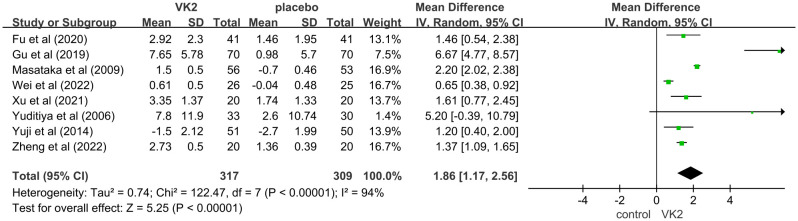
Effect of VK2 on OC. Axes are zoomed to enhance readability where very wide and very narrow CIs co-occur. Arrowheads denote CIs truncated by the axis limits; full-range plots are provided in **Supplementary Figures S2**.

This meta-analysis evaluated the effect of vitamin K2 (VK2) on bone metabolism, with osteocalcin (OC) included as a marker of bone formation. A total of 8 randomized controlled trials (RCTs) met the inclusion criteria, involving 626 participants (317 in the VK2 group and 309 in the control group). Pooled analysis demonstrated that VK2 significantly increased serum OC levels compared with placebo (mean difference [MD] = 1.86, 95% confidence interval [CI]: 1.17–2.56, p < 0.00001), indicating a potential promotive effect of VK2 on bone formation. Although substantial heterogeneity was observed (I² = 94%), the direction of effect remained consistent across all included studies (**Figure 3**).

In the overall analysis of osteocalcin (OC), substantial heterogeneity was observed (I² = 94%). To explore potential sources of heterogeneity, subgroup analyses were conducted based on intervention dose, control type, and duration of intervention. The findings indicated that these factors contributed notably to between-study variability. Specifically, a greater effect size was observed in the 45 mg/day subgroup compared to the 15 mg/day subgroup, suggesting a dose-dependent effect of VK2 on OC. Additionally, heterogeneity remarkably decreased when the control group included bisphosphonates in combination with calcium and vitamin D (I² = 0%), indicating that variations in background therapy may account for part of the heterogeneity. Moreover, studies with intervention durations of ≥6 months demonstrated more consistent outcomes (I² = 0%), implying that the effect of VK2 may require sufficient treatment duration to stabilize, while shorter interventions resulted in greater variability.

**Figure 4:**
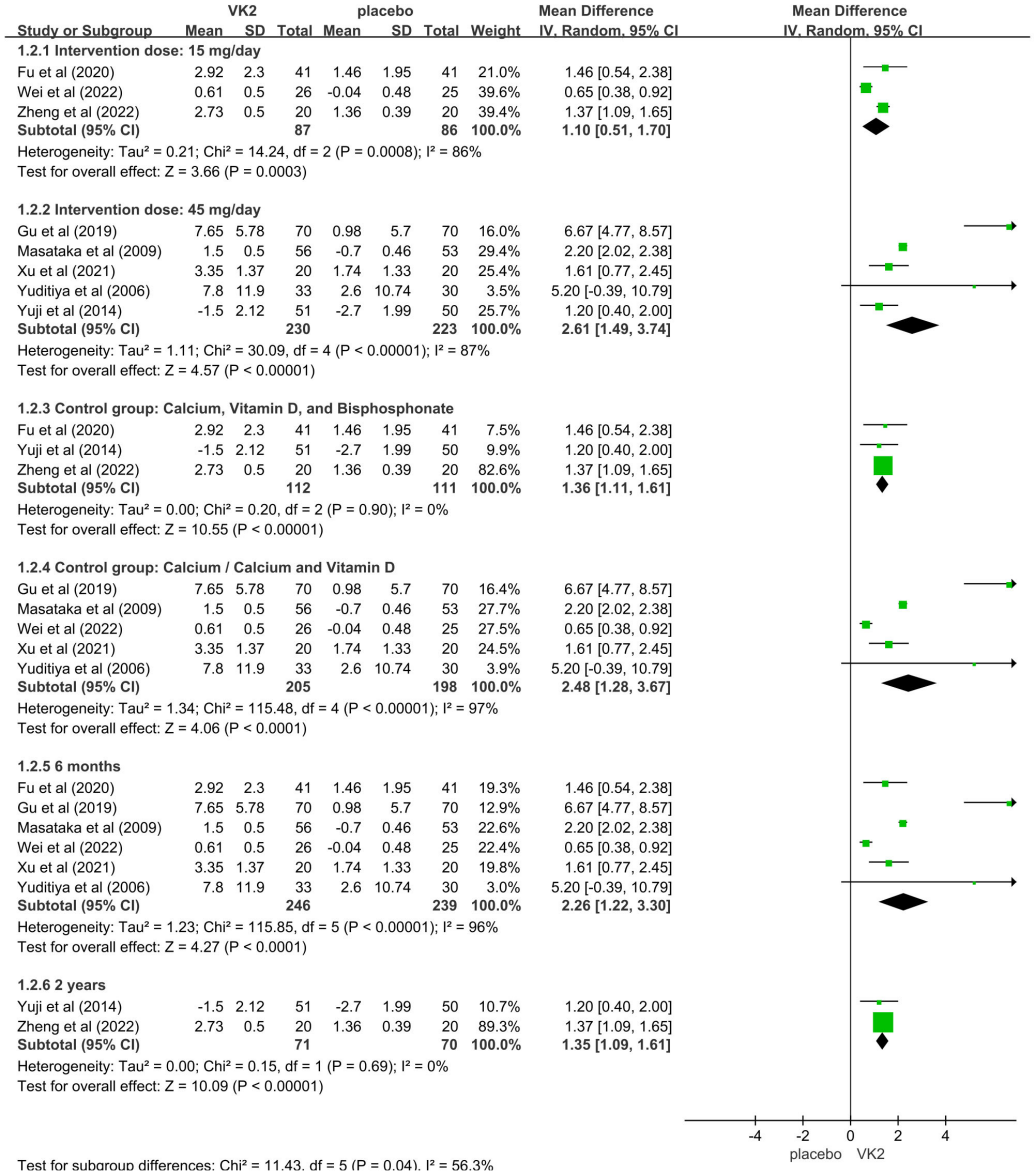
Subgroup analyses for osteocalcin (OC) Axes are zoomed to enhance readability where very wide and very narrow CIs co-occur. Arrowheads denote CIs truncated by the axis limits; full-range plots are provided in **Supplementary Figures S3**.

Sensitivity analysis showed that sequential removal of individual studies did not substantially alter the pooled effect size or the direction of effect, demonstrating the robustness of the findings. Although heterogeneity was present in the overall analysis, it was largely explained by subgroup analyses, and the stability of the pooled results supports the reliability of the conclusion that VK2 exerts a beneficial effect on OC levels (**Figure 4**).

In the OC and OC subgroup analyses, some studies had extremely wide CIs whereas others were very narrow, which cannot be accommodated on a single x-axis without loss of readability. Accordingly, the main forest plot displays a zoomed range; full-range, untruncated plots are available in **Supplementary Figures S2, S3**.

#### 3.4.2 ucOC(ng/mL)

**Figure 5:**
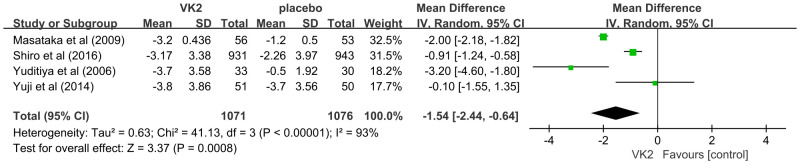
Effect of VK2 on ucOC.

The effect of vitamin K2 on undercarboxylated osteocalcin (ucOC), a biomarker of vitamin K status and γ-carboxylation efficiency, was assessed in four randomized controlled trials involving a total of 2,147 participants (1,071 in the VK2 group and 1,076 in the control group). The pooled analysis demonstrated that VK2 significantly reduced serum ucOC levels compared with placebo (weighted mean difference [WMD] = –1.54, 95% confidence interval [CI]: –2.44 to –0.64, p = 0.0008), indicating enhanced osteocalcin carboxylation and improved bone metabolic activity. Considerable heterogeneity was observed across the included studies (I² = 93%). Owing to the limited number of studies, subgroup analysis could not be performed; however, sensitivity analysis revealed that sequential exclusion of individual studies did not materially alter the effect estimate, confirming the robustness and stability of the pooled results despite heterogeneity (**Figure 5**).

#### 3.4.3 CTX(ng/mL)

**Figure 6:**
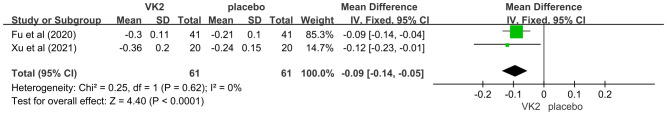
Effect of VK2 on CTX.

The effect of vitamin K2 on the bone resorption marker C-terminal telopeptide of type I collagen (CTX) was evaluated in two randomized controlled trials including 122 participants (61 in the VK2 group and 61 in the placebo group). Meta-analysis showed that VK2 slightly reduced serum CTX levels compared with placebo (MD = –0.09, 95% CI –0.14 to –0.05, p < 0.0001), with no statistical heterogeneity (I² = 0%). However, the magnitude of this reduction was small, suggesting a limited effect of VK2 on inhibiting bone resorption. Furthermore, since only two studies were included and both were conducted in Chinese populations, the generalizability of this result is limited and should be interpreted with caution (**Figure 6**).

#### 3.4.4 NTX

**Figure 7:**
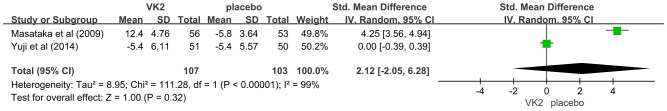
Effect of VK2 on NTX.

Two studies reported NTX levels but used different measurement forms: one used urinary NTX (U-NTX, nmol/mmol Cr), while the other used serum NTX (S-NTX, nmol BCE/L). Therefore, standardized mean difference (SMD) was applied for data pooling. The meta-analysis showed no significant difference in NTX levels between the VK2 and placebo groups (SMD = 2.12, 95% CI –2.05 to 6.28, p = 0.32), and heterogeneity was extremely high (I² = 99%). These results indicate that vitamin K2 did not produce a consistent effect on NTX, whether measured in urine or serum (**Figure 7**).

#### 3.4.5 BAP(μg/L)

**Figure 8:**
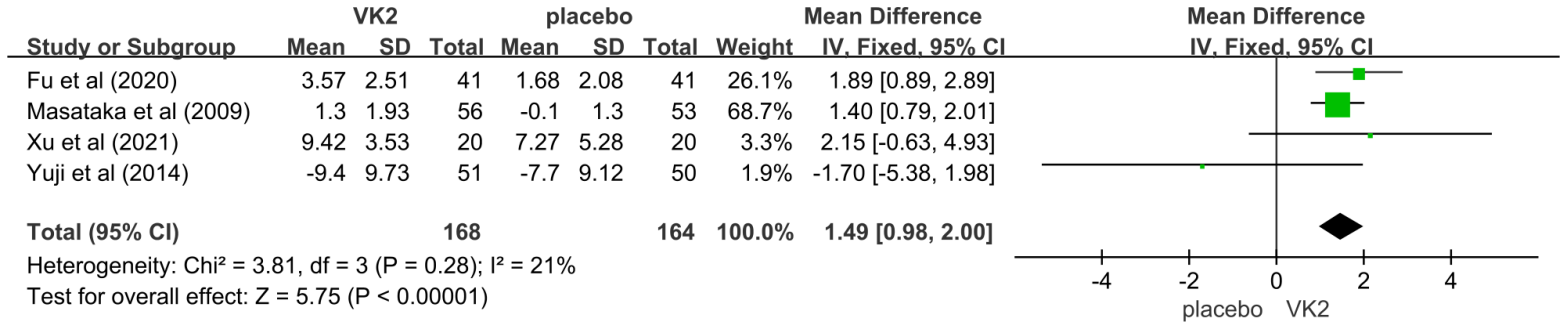
Effect of VK2 on BAP.

The effect of vitamin K2 on bone formation activity was further assessed using bone-specific alkaline phosphatase (BAP). Four randomized controlled trials involving 332 participants (168 in the VK2 group and 164 in the placebo group) were included. The pooled analysis demonstrated that vitamin K2 significantly increased serum BAP levels compared with placebo (mean difference [MD] = 1.49, 95% confidence interval [CI] 0.98 to 2.00, p < 0.00001), indicating a stimulatory effect of VK2 on bone formation. Heterogeneity across studies was low and acceptable (I² = 21%), and thus, a fixed-effects model was applied (**Figure 8**).

#### 3.4.6 TRAP(U/L)

**Figure 9:**
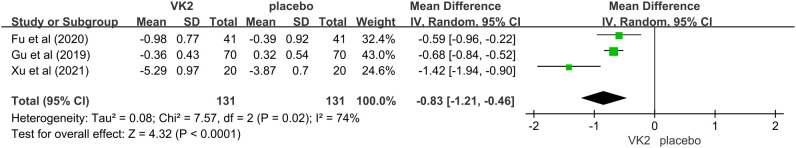
Effect of VK2 on TRAP.

The effect of vitamin K2 on tartrate-resistant acid phosphatase (TRAP), a biochemical marker of osteoclastic bone resorption, was reported in three randomized controlled trials comprising 262 participants (131 in the VK2 group and 131 in the placebo group). The pooled analysis demonstrated that vitamin K2 significantly reduced TRAP levels compared with placebo (mean difference [MD] = –0.83, 95% confidence interval [CI]: –1.21 to –0.46, p < 0.0001), suggesting a potential inhibitory effect on osteoclast-mediated bone resorption. Moderate heterogeneity was detected (I² = 74%), and therefore a random-effects model was adopted. Despite the variability among studies, the direction of effect was consistent, supporting a suppressive role of VK2 on bone resorption (**Figure 9**).

#### 3.4.7 Publication bias assessment results

**Figure 10:**
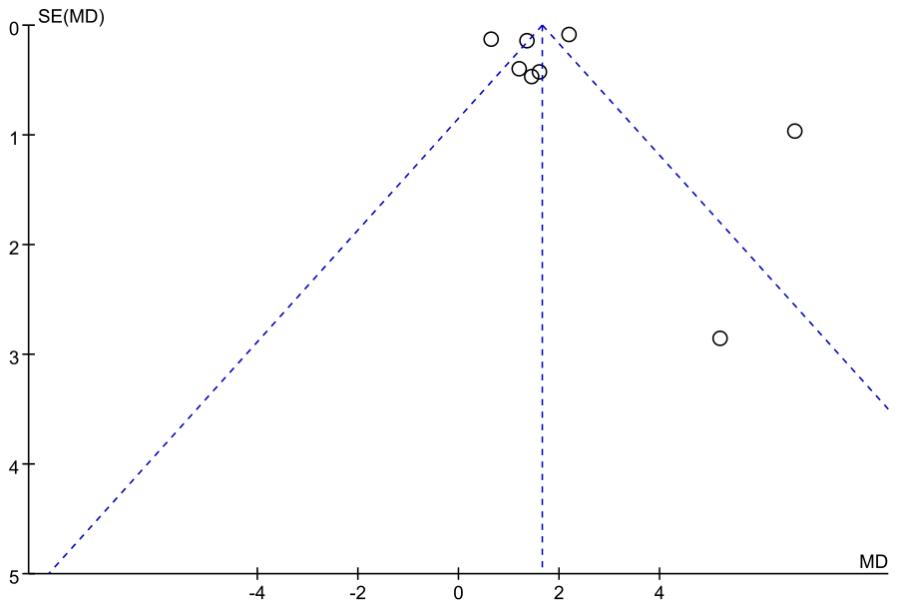
Funnel plot of OC.

Assessment of publication bias for the osteocalcin (OC) outcome was conducted using a funnel plot. The distribution of studies showed a slight degree of asymmetry, with several data points deviating from the central line of the pooled effect estimate. In particular, a few studies appeared in the lower right region of the plot, which may reflect a potential small-study effect. Although this pattern does not provide definitive evidence of publication bias, it suggests that its influence cannot be fully excluded, and therefore the pooled results for OC should be interpreted with appropriate caution (**Figure 10**).

## 4 Discussion

In summary, vitamin K_2_ supplementation was associated with favorable changes in bone metabolism. Our meta-analysis demonstrated increases in osteocalcin (OC) and bone-specific alkaline phosphatase (BAP) alongside reductions in undercarboxylated osteocalcin (ucOC), consistent with enhanced γ-carboxylation and formation-side activity. Effects on resorption were modest: tartrate-resistant acid phosphatase (TRAP) decreased, whereas C-telopeptide (CTX) and N-telopeptide (NTX) showed no significant changes (urinary U-NTX and serum S-NTX).

Consistent with prior evidence, vitamin K lowered ucOC without affecting BAP, NTX, or BMD in healthy postmenopausal North American women (33). In a 3-year placebo-controlled add-on trial with universal calcium/vitamin D (Ca/D), MK-7 (375 μg/day) improved osteocalcin carboxylation but did not alter bone turnover markers, BMD, or microarchitecture (18). By contrast, another 3-year trial reported that low-dose MK-7 (180 μg/day) attenuated age-related BMD loss while improving vitamin K status. This pattern suggests that formation-side changes can translate into structural preservation with longer follow-up (17).

Mechanistically, vitamin K_2_ appears to act predominantly on osteoblasts. As a cofactor for γ-glutamyl carboxylase (GGCX), it promotes γ-carboxylation of vitamin K–dependent proteins—most notably osteocalcin—thereby increasing hydroxyapatite binding, matrix maturation, and mineralization (13, 34, 35). The observed decrease in TRAP is compatible with downstream modulation of osteoclast activity via SXR/PXR signaling, up-regulation of osteoprotegerin (OPG), and attenuation of RANKL/NF-κB (35–38). Taken together, these features indicate a formation-dominant profile with limited direct antiresorptive action—consistent with the null effects for CTX/NTX.

Three factors likely explain the frequent null findings for CTX/NTX. First, formation-dominant biology under K_2_ renders resorption markers less sensitive over short- to mid-term follow-up (39). Second, pre-analytical variability dilutes between-group differences: serum β-CTX shows pronounced circadian and feeding-related oscillations and depends on specimen matrix/assay; harmonized morning, fasting, matrix-consistent protocols are therefore recommended, and β-CTX variability exceeds that of formation markers such as PINP (40–42). Third, Ca/D co-interventions suppress resorption. They can mask small antiresorptive effects of K_2_ (18, 33, 43). Randomized trials in Denmark and the United States reported large reductions in ucOC with MK-7 or phylloquinone/MK-4. They did not find consistent changes in CTX/NTX when all participants received Ca/D (18, 33, 43). Ca/D-fortified dairy with K_1_ or MK-7 improved vitamin K status. Changes in resorption markers were small or inconsistent (44). Collectively, these points provide a coherent explanation for CTX/NTX null results under K_2_ (45).

Between-study heterogeneity was substantial (I² > 90% for most indicators). Random-effects models and standardized mean differences did not remove it. Vitamer, dose, comparator, and treatment duration varied across trials. These differences likely drove the heterogeneity. Risk-of-bias assessments showed methodological uncertainties, such as inadequate blinding. These issues may inflate effect estimates.

This review has several strengths. It followed PRISMA and was prospectively registered on PROSPERO. It used comprehensive searches across Chinese and English databases. It focused on biochemical outcomes in postmenopausal osteoporosis. It also applied rigorous risk-of-bias assessment.

Limitations should be noted. The number of included studies was small. Several had small samples and short follow-up, mostly 6 months. These features reduced statistical power and limited long-term inference. Differences in bone marker assay methods contributed to heterogeneity. Potential confounding, including dietary vitamin K intake and concomitant medications, was not consistently controlled. The link between biochemical changes and fracture risk needs confirmation in long-term trials. Inclusion of studies in multiple languages may have introduced measurement and reporting variability.

Future work should prioritize large, high-quality randomized trials that last 1–2 years. Testing should be standardized, especially for osteocalcin species (cOC/ucOC). Reporting should include baselines, changes, endpoints, and variability metrics. Studies should define dose–response and optimal regimens for MK-4 versus MK-7. Long-term trials that integrate BMD and fracture endpoints are needed to establish clinical significance.

## 5 Conclusions

The findings indicate that vitamin K2 supplementation significantly improves vitamin K status in osteoporotic patients, manifested by markedly reduced uncarboxylated osteocalcin (ucOC) levels and elevated total osteocalcin (OC) levels. Vitamin K2 also confers benefits by reducing the bone resorption marker TRAP and elevating 25(OH)D concentrations. However, its effects on other key synthesis markers (PINP, BAP) and resorption markers (CTX, NTX) did not reach statistical significance. Although conclusions are limited by the number of included studies, substantial heterogeneity, and potential bias, these findings support the potential role of vitamin K2 in improving bone metabolism by enhancing osteocalcin carboxylation and possibly modulating bone turnover processes. Low-carboxylated osteocalcin may serve as an effective surrogate marker for assessing vitamin K2 bioactivity. Future long-term, high-quality studies are needed to validate whether improvements in these biochemical markers translate to clinical benefits—namely, increased bone mineral density.

## Data Availability

The original contributions presented in the study are included in the article/**Supplementary Material**. Further inquiries can be directed to the corresponding author.
