# Supplementary figures and images for: The effect of vitamin K2 supplementation on bone turnover biochemical markers in postmenopausal osteoporosis patients: a systematic review and meta-analysis
Source: Front Endocrinol (Lausanne). 2025 Nov 5;16:1703116. doi: 10.3389/fendo.2025.1703116 (PMC12626859; doi:10.3389/fendo.2025.1703116)

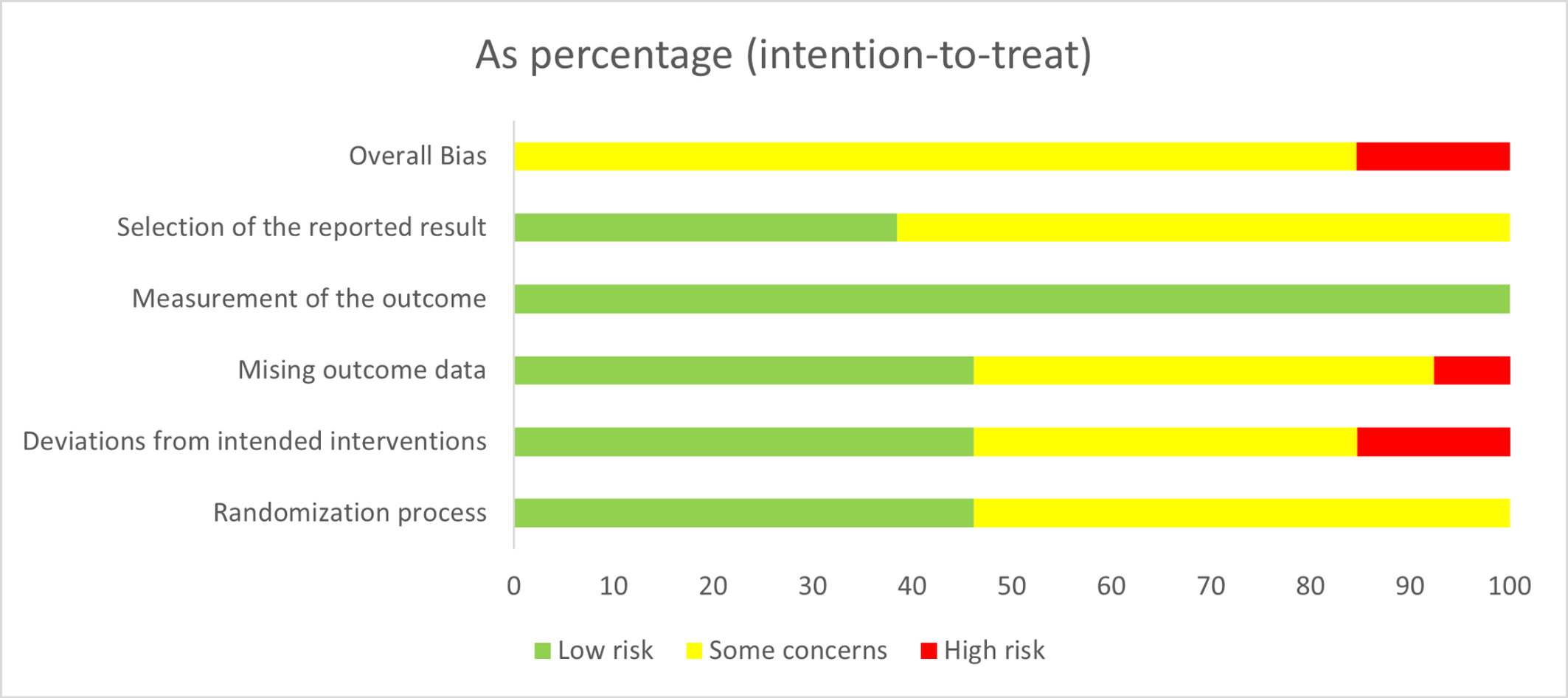

Supplement: Supplementary file 1 [file Image1.tif]

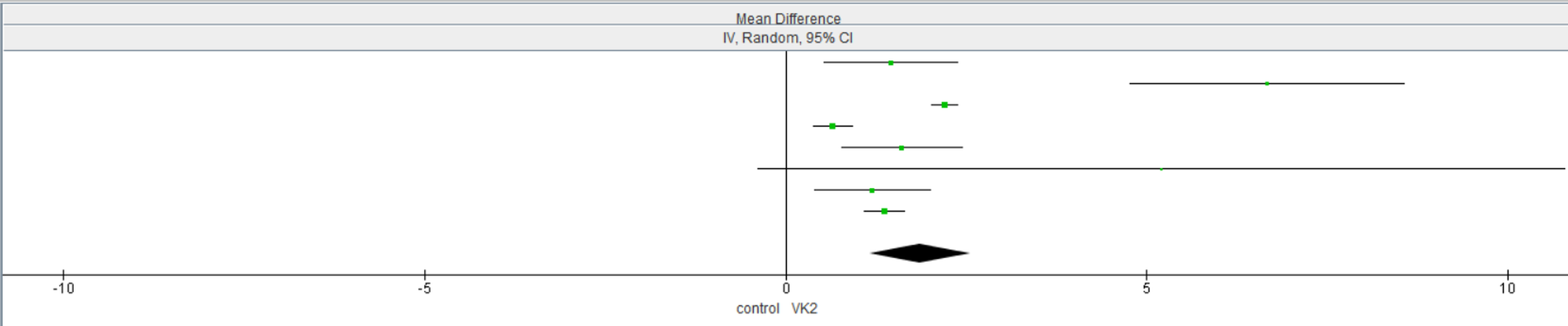

Supplement: Supplementary file 2 [file Image2.tif]

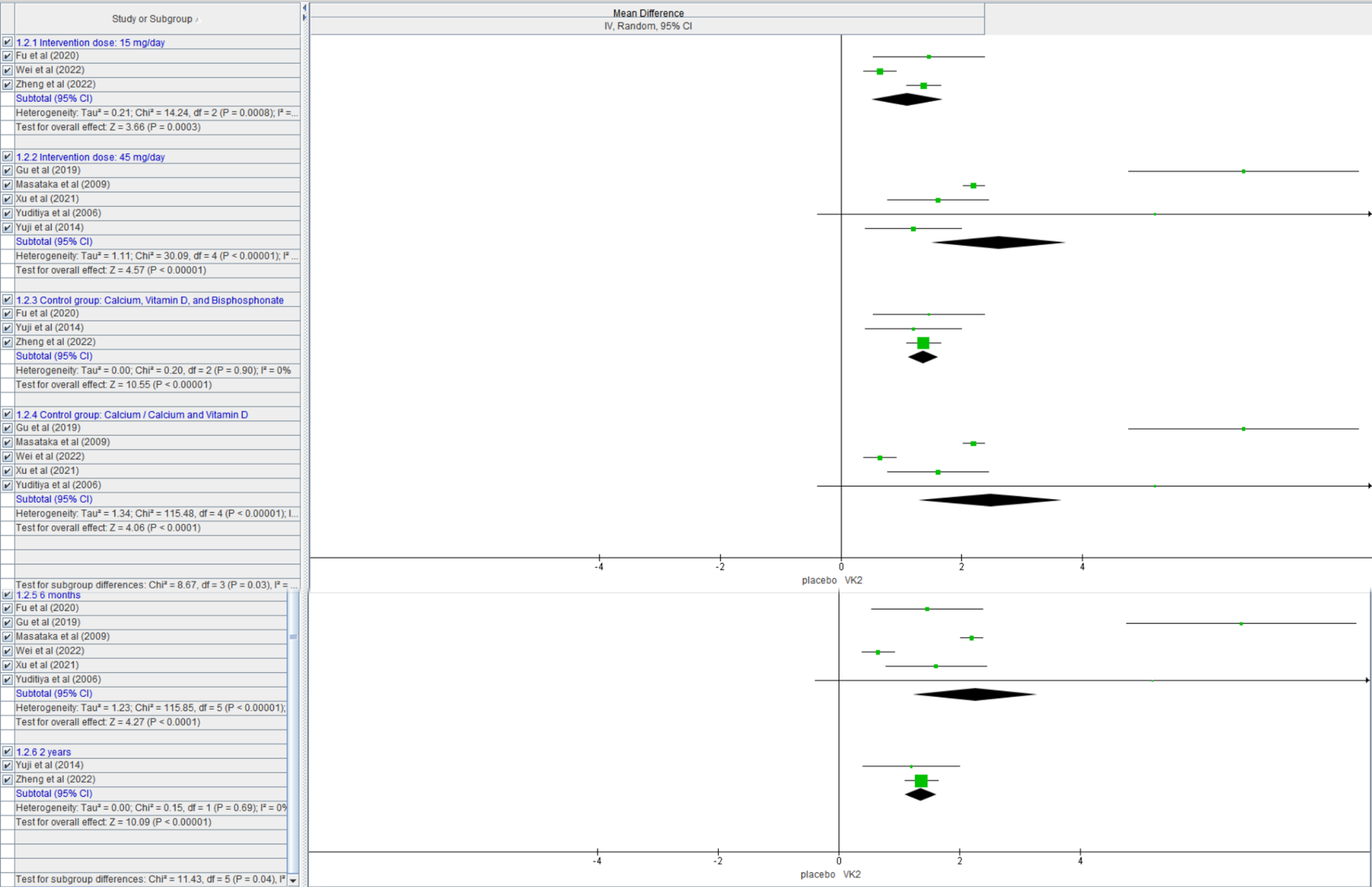

Supplement: Supplementary file 3 [file Image3.tif]

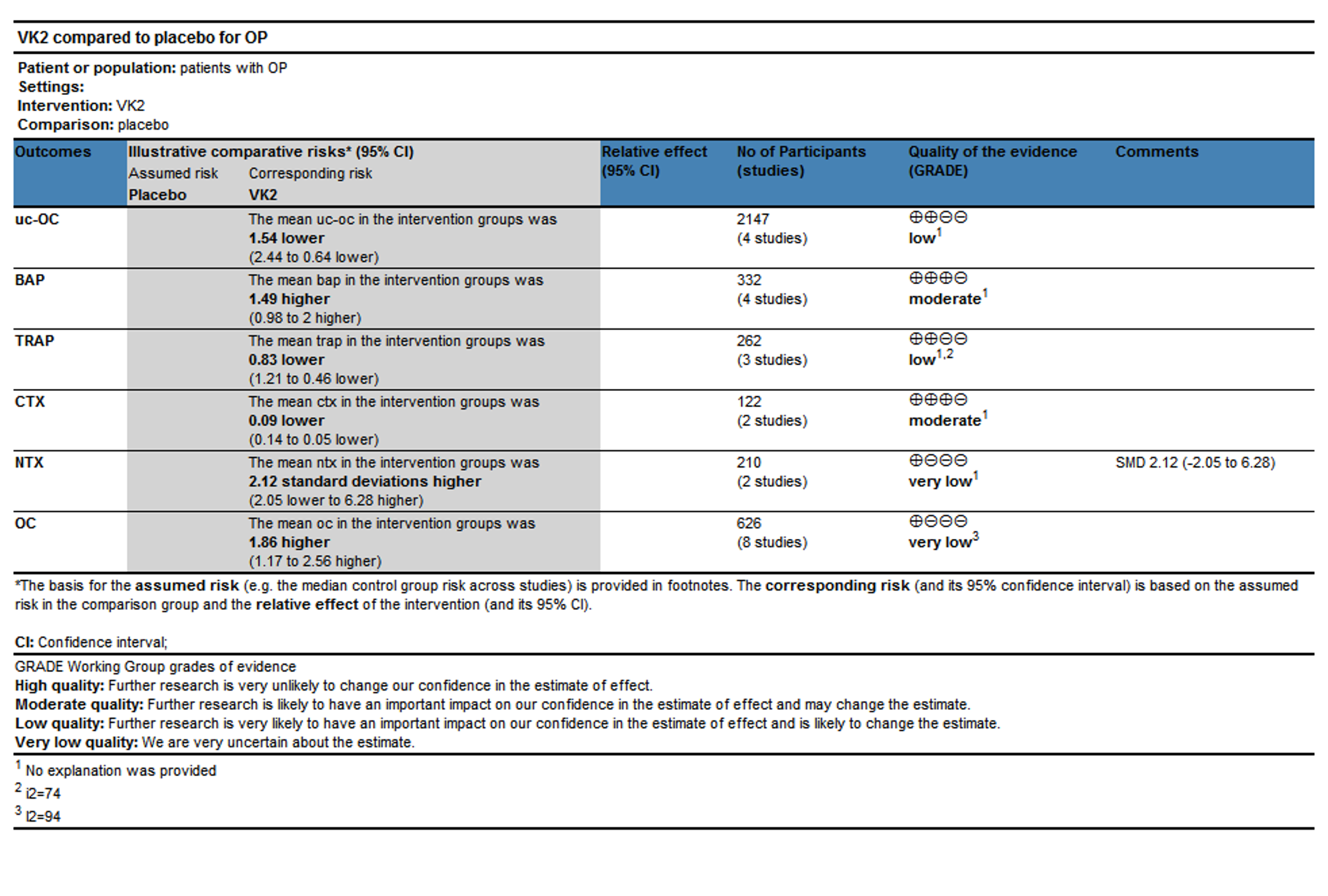

Supplement: Supplementary file 4 [file Image4.tif]
