# Supplementary material for: The effect of vitamin K2 supplementation on bone turnover biochemical markers in postmenopausal osteoporosis patients: a systematic review and meta-analysis
Source: Front Endocrinol (Lausanne). 2025 Nov 5;16:1703116. doi: 10.3389/fendo.2025.1703116 (PMC12626859; doi:10.3389/fendo.2025.1703116)
Supplement: Supplementary file 5 [file Table1.docx]

- PubMed（197）

#1

("Vitamin K2"[tiab] OR "Menatetrenone"[tiab] OR "MK-4"[tiab] OR "Menaquinone 4"[tiab] OR "Menaquinone-4"[tiab] OR "MK-7"[tiab] OR "Menaquinone 7"[tiab] OR "Menaquinone-7"[tiab] OR "Menaquinone*"[tiab] OR "vitamin K 2"[tiab]) OR ("Vitamin K 2"[Mesh] OR "Menaquinone"[Mesh])

# 2

("Osteoporosis"[tiab] OR "Osteoporoses"[tiab] OR "Osteoporotic"[tiab] OR "Bone Loss"[tiab] OR "Bone Atrophy"[tiab] OR "Bone Density"[tiab] OR "Bone Mineral Density"[tiab] OR "BMD"[tiab] OR "Fracture"[tiab] OR "Fractures"[tiab] OR "vertebral fracture"[tiab] OR "hip fracture"[tiab] OR "nonvertebral fracture"[tiab] OR "non-vertebral fracture"[tiab] OR "bone fracture"[tiab]) OR ("Osteoporosis"[Mesh] OR "Bone Density"[Mesh] OR "Fractures, Bone"[Mesh] OR "Osteoporotic Fractures"[Mesh])

#3

(random*[tiab] OR trial[tiab] OR groups[tiab] OR "controlled"[tiab] OR "clinical study" OR "clinical trial")

(#1) AND (#2) AND (#3)

- Wos（165）

# 1

TS=(("vitamin k2" OR menatetrenone OR mk4 OR "mk-4" OR "menaquinone 4" OR "menaquinone-4" OR mk7 OR "mk-7" OR "menaquinone 7" OR "menaquinone-7" OR menaquinone*))

# 2

TS=( (osteoporosis OR osteoporoses OR osteoporotic OR "bone loss" OR "bone atrophy" OR "bone density" OR "bone mineral density" OR bmd OR fracture OR fractures OR "vertebral fracture" OR "hip fracture" OR "nonvertebral fracture" OR "non-vertebral fracture" OR "bone fracture") )

#3

TS=(random* OR RCT OR "randomized controlled trial" OR placebo OR "clinical trial" OR trial OR group*)

#1 AND #2 AND #3

Embase（78）

1. (vitamin k2 or menatetrenone or mk4 or mk-4 or menaquinone 4 or menaquinone-4 or mk7 or mk-7 or menaquinone 7 or menaquinone-7 or menaquinone*).ti,ab,kw.

2. exp menaquinone/

3. 1 or 2

4. (osteoporosis or osteoporoses or osteoporotic or bone loss or bone atrophy or bone density or bone mineral density or bmd or fracture or fractures or vertebral fracture or hip fracture or nonvertebral fracture or non-vertebral fracture or bone fracture).ti,ab,kw.

5. exp osteoporosis/ or exp bone density/ or exp fracture/ or exp osteoporotic fracture/

6. 4 or 5

7. 3 and 6

- Cochrane Library（139）

#1: [mh "Vitamin K 2"] or [mh "Menaquinone"]

#2: (vitamin k2 or menatetrenone or mk4 or mk-4 or menaquinone 4 or menaquinone-4 or mk7 or mk-7 or menaquinone 7 or menaquinone-7 or menaquinone*):ti,ab,kw

#3: #1 or #2

#4: [mh Osteoporosis] or [mh "Bone Density"] or [mh "Fractures, Bone"] or [mh "Osteoporotic Fractures"]

#5: (osteoporosis or osteoporoses or osteoporotic or bone loss or bone atrophy or bone density or bone mineral density or bmd or fracture or fractures or vertebral fracture or hip fracture or nonvertebral fracture or non-vertebral fracture or bone fracture):ti,ab,kw

#6: #4 or #5

#7: #3 and #6

- CNKI (211)

SU= ((vitamin K2 OR vitamin K2 OR tetraoxanthin OR MK-4 OR MK4 OR 'tetraoxanthin-4' OR tetraoxanthin-4 OR MK-7 OR MK7 OR 'tetraoxanthin-7' OR tetraoxanthin-7 OR 'vitamin K2') AND (osteoporosis OR osteoporosis OR reduced bone mass OR bone density OR bone mineral density OR BMD OR fracture OR vertebral fracture OR hip fracture OR femoral neck fracture OR non-vertebral fracture))

- Wanfang (161)

("vitamin K2" OR "vitamin K2" OR "tetraoxanthin" OR "MK-4" OR "MK4" OR "oxanthin-4" OR "oxanthin-4" OR "MK-7" OR "MK7" OR "oxanthin-7" OR "oxanthin-7" OR "vitamin K2") AND ("osteoporosis" OR "osteoporosis" OR "bone loss" OR "bone density" OR "bone mineral density" OR "BMD" OR "fracture" OR "vertebral fracture" OR "hip fracture" OR "femoral neck fracture" OR "non-vertebral fracture")

- China Biomedical Literature Database (87)

#1: ("Vitamin K2" [Common Field: Smart] OR "Vitamin K2" [Common Field: Smart] OR "Tetramethoxybenzoquinone" [Common Field: Smart] OR "MK-4" [Common Field: Smart] OR "MK4" [Common Field: Smart] OR "Methoxybenzoquinone-4" [Common Field: Smart] OR "Methoxybenzoquinone 4" [Common Field: Smart] OR "MK-7" [Common Field: Smart] OR "MK7" [Common Field: Smart] OR "Methoxybenzoquinone-7" [Common Field: Smart] OR "Methoxybenzoquinone 7" [Common Field: Smart] OR "Vitamin K2" [Common Field: Smart])

#2: ("Osteoporosis" [Common Field: Intelligent] OR "Osteoporosis" [Common Field: Intelligent] OR "Bone Loss" [Common Field: Intelligent] OR "Bone Density" [Common Field: Intelligent] OR "Bone Mineral Density" [Common Field: Intelligent] OR "BMD" [Common Field: Intelligent] OR "Fracture" [Common Field: Intelligent] OR "Vertebral Fracture" [Common Field: Intelligent] OR "Hip Fracture" [Common Field: Intelligent] OR "Femoral Neck Fracture" [Common Field: Intelligent] OR "Non-Vertebral Fracture" [Common Field: Intelligent])

#3: #1 AND #2
